# Interprofessional Education with Graduate Students in Dietetics and Speech-Language Pathology: A Sequential-Explanatory Investigation

**DOI:** 10.1055/a-2914-5938

**Published:** 2026-07-28

**Authors:** David M. Rehfeld, Jane Mertz Garcia, Taylor Nading, Kelly Whitehair

**Affiliations:** 5308 School of Health Sciences, Kansas State UniversityManhattanKansasUnited States

**Keywords:** dysphagia, interprofessional education, interprofessional collaborative practice, interprofessional practice

## Abstract

Interprofessional education (IPE) prepares students to engage in interprofessional collaborative practice (IPCP), which is essential for managing complex clinical conditions. This mixed methods study examined the impact of a dysphagia-focused IPE sequence involving pre-service speech-language pathologists (SLPs) and registered dietitians (RDs) on their perceptions of collaboration and understanding of professional roles. A total of 31 graduate students (23 SLP, 8 RD) participated in two case-based IPE sessions embedded within existing coursework. Quantitative data were collected using the Student Perceptions of Interprofessional Clinical Education–Revised (SPICE-R) while qualitative data were gathered through open-ended reflections and analyzed using reflexive thematic analysis. Results indicated statistically significant improvements on the SPICE-R alongside significant increase in participants' perceived understanding of SLP and RD roles in dysphagia management. Qualitative findings illustrated how these changes occurred, revealing shifts from generalized descriptions of collaboration to more specific understandings of roles, communication, and shared responsibility for patients. There was strong convergence between the quantitative and qualitative strands, with qualitative themes elaborating on the processes underlying improvements in students' perceptions of IPCP. These findings suggest targeted IPE experiences embedded within existing coursework can meaningfully influence trainees' perceived readiness for dysphagia care.

**Learning Outcomes** : As a result of this activity, the reader will be able to:

- Explain the roles and responsibilities of speech-language pathologists and registered dietitians in dysphagia management.
- Summarize how structured interprofessional education experiences influence graduate students' perceptions of collaboration and professional roles.
- Discuss how case-based interprofessional education activities can support collaborative clinical reasoning in dysphagia care.

## Introduction

Delivering safe, effective, and patient-centered care for individuals with complex medical conditions requires coordinated clinical decision-making across healthcare professions (Herath et al., 2017). Clinical decisions related to swallowing disorders, also known as dysphagia, frequently require simultaneous consideration of swallowing safety, nutritional adequacy, medical stability, and quality of life—domains that span professional expertise and cannot be optimally addressed by any single profession in isolation (McGinnis et al., 2019). Speech-language pathologists (SLPs) and registered dietitians (RDs) hold interdependent responsibilities in dysphagia care, particularly when clinical decisions require balancing aspiration risk with nutritional status and patient preferences. As such, effective dysphagia management depends not only on profession-specific competence, but also on the ability to engage in collaborative clinical reasoning, negotiate shared responsibility with potential tension between professional priorities, and communicate across professional scopes of practice. This need for integrated pre-professional training is further underscored by recent efforts to better acknowledge the impact of nutrition on overall health outcomes during the training of future healthcare professionals (U.S. Department of Health and Human Services, 2026).

## Background

Despite the inherently interprofessional nature of dysphagia care, pre-professional training in many allied health programs remains largely profession-specific due to the specialized knowledge and skills needed for entry into professional practice (American Speech-Language-Hearing Association, 2020). Training in professions such as speech-language pathology and dietetics prioritizes the development of profession-specific knowledge and clinical skills, often providing limited structured opportunities for students to engage in shared decision-making with other professions before entering independent practice (van der Weerd et al., 2025). As a result, learners may develop strong procedural expertise within their own scope of practice while lacking experiential understanding of how their decisions intersect with those of colleagues in clinical contexts. Effective dysphagia care, however, depends on interprofessional collaborative practice (IPCP): the coordinated, team-based care through which professionals integrate their distinct expertise around shared patient goals.

Interprofessional education (IPE) has emerged as a primary strategy to develop readiness for IPCP in pre-service training. The World Health Organization (2010) defines IPE as learning experiences in which students from two or more professions learn with, from, and about one another to improve collaboration and health outcomes. By bringing trainees from different professions into shared educational activities, well-designed IPE provides the structured opportunities for engaging with other professions that professional training programs may otherwise leave underdeveloped. In doing so, IPE can support the development of the IPCP competencies emerging professionals need to deliver effective team-based dysphagia care, including values and ethics, roles and responsibilities, interprofessional communication, and teams and teamwork (Interprofessional Education Collaborative, 2023).

Substantial research indicates that IPE is generally associated with improvements in trainees' attitudes toward collaboration, understanding of professional roles, and perceived readiness for team-based practice (Spaulding et al., 2021; Saragih et al., 2023; Patel et al., 2025). A recent systematic review and meta-analysis also found that IPE interventions consistently yield positive effects on professional role clarity and collaborative attitudes (Antony et al., 2025). These outcomes correspond to the early levels of Kirkpatrick and Kirkpatrick's (2016) evaluation framework, which capture changes in trainee perceptions and knowledge. Much of this evidence, however, documents the occurrence of such changes without examining how they develop as students learn together, leaving the processes underlying these early-level gains comparatively underexplored in the existing literature.

Implementing IPE effectively in pre-professional healthcare training programs, however, requires careful attention to the time and resources these activities demand. For example, Nandamudi and colleagues (2023) implemented a full-day IPE in-person activity conducted in 18 to 20 person groups that was preceded by “multiple short (10–15 min) instructional videos” (p. 2) and followed by virtual laboratory assignments of an unreported length, representing a significant investment of both time and other resources for faculty and students. In contrast, Page et al. (2023) investigated a 2-hour virtual IPE activity with 11 to 12 participants per group but found unequal preparation across professions and large group sizes negatively impacted students' perceptions of the experience. Because IPE often relies on simulations to deliver realistic learning opportunities for students, it also requires structured pre-briefing and debriefing activities to prepare trainees for collaboration, establish psychological safety, and support guided reflection to consolidate learning and encourage transfer to future performance (Cheng et al., 2021; Chan et al., 2023; Penn et al., 2023; Marita Christina Susanne et al., 2026). Given the wide variety in programming reported in the literature (Saragih et al., 2023), attention to these logistical demands is necessary to ensure that IPE experiences are effective and meaningful for learners.

Although IPE, IPCP, and interprofessional practice (IPP) are all referenced in the current certification standards for SLPs (American Speech-Language-Hearing Association, 2020), the current standards for RDs only refer to a need for trainees to develop competence in functioning as a member of interprofessional teams (Accreditation Council for Education in Nutrition and Dietetics, 2025). Furthermore, there is limited research on IPE's implementation between specific allied health dyads such as speech-language pathology and dietetics (e.g., Reeves et al., 2017; Boshoff et al., 2020). Within dysphagia education specifically, emerging investigations into IPE are indicating promising outcomes. For example, simulation-based IPE initiatives involving SLP students have reported increased confidence and perceived collaborative competence following interprofessional case experiences (Weir-Mayta et al., 2020). Similarly, dysphagia-focused IPE experiences involving nursing, nutrition, and speech-language pathology students have demonstrated significant improvements in self-efficacy across Interprofessional Education Collaborative (IPEC) competencies following structured case-based collaboration (Tilmon et al., 2024). IPE between medical and speech-language pathology students in dysphagia management has likewise been associated with enhanced understanding of professional roles and greater appreciation of collaborative care (Kelly et al., 2021).

Despite these promising findings, however, most dysphagia-focused IPE studies rely primarily on pre–post self-efficacy or attitudinal measures and single-method designs. Although such findings provide important evidence of short-term perceptual gains, they offer limited insight into how learners' role conceptualizations, boundary negotiations, and collaborative reasoning processes evolve during shared clinical tasks. As emphasized in recent reviews of IPE scholarship, there remains a need for theoretically grounded qualitative investigations that clarify the mechanisms through which interprofessional learning occurs during these experiences, particularly within allied health contexts (Antony et al., 2025). Understanding the mechanisms underlying IPE is especially important in high-risk areas of practice such as dysphagia, where siloed expertise may contribute to fragmented decision-making and poor patient outcomes. Mixed methods approaches are well-suited to address this aim, as they offer a more comprehensive account of how IPE influences both measurable perception change and the processes that underlie emerging interprofessional competencies. However, current mixed-methods work on IPE often depends on datasets in which participants have not consistently contributed to both the quantitative and qualitative strands of a study, limiting the integration that mixed-methods designs are intended to achieve (e.g., Kelly et al., 2021).

Studies of IPE incorporating qualitative data collection must also consider sociocultural perspectives that conceptualize learning as active participation in shared experiences as opposed to perspectives that emphasize individual effort (Lees & Meyer, 2011; Roberts & Kumar, 2015). From this perspective, learning from IPE occurs through engagement in authentic clinical tasks that require engagement with other emerging professionals from different professional backgrounds. Collaborative clinical reasoning—defined as the process by which professionals jointly interpret clinical information and reconcile profession-specific priorities (Kiesewetter et al., 2017)—provides one such lens for understanding how students reason together during dysphagia management. Participation in such collaborative learning activities may also contribute to early interprofessional socialization and professional identity development as learners situate their profession-specific expertise within an interprofessional framework (Khalili et al., 2013; Cruess et al., 2014; Grymonpre et al., 2023).

## Purpose of the Study

The present study addresses gaps in the existing literature by evaluating a concise, dysphagia-focused IPE sequence embedded within graduate speech-language pathology and dietetics coursework using a sequential explanatory mixed methods design. Student perceptions of IPCP and roles related to dysphagia care were explored quantitatively while qualitative analyses were used to examine trainees' descriptions of professional boundaries, responsibilities, and clinical reasoning in dysphagia care.

## Research Questions

1. How does participation in an IPE sequence embedded in dysphagia coursework influence SLP and RD students' understanding of professional roles and collaborative responsibilities within dysphagia management?
2. How do SLP and RD students describe changes in their understanding of profession-specific roles, boundaries, and shared responsibilities in dysphagia care following participation in the IPE sequence?
3. How do students' written reflections elaborate on quantitative changes by illustrating how a dysphagia-focused IPE experience shaped their collaborative reasoning and emerging professional identities?

## Methods

### Study Design and Context

This study was reviewed by the Kansas State University Institutional Review Board (IRB #12363) and used a sequential-explanatory mixed methods design (Creswell & Plano Clark, 2018) to evaluate the impact of an IPE sequence on graduate students' understanding of professional roles and interprofessional practice in dysphagia management.

### Participants

Participants included 31 graduate students enrolled in either a master's-level SLP or RD program at Kansas State University during the Fall 2024 semester. The sample included 23 SLP students and 8 RD students who completed the IPE sequence as part of a required graduate course in dysphagia or clinical nutrition. SLP students enrolled in the dysphagia course were at various points in their curriculum prior to full-time external clinical placements with minimal adult medical clinical experiences. In contrast, RD students were enrolled in a practicum course and had completed placements in community and management settings with one acute care placement remaining the semester after the present study occurred. All students in both programs had completed appropriate prerequisites for dysphagia content in their respective field of study. Study information was reviewed with students by a member of the research team during a scheduled class meeting without instructors present. All 23 eligible SLP students and 8 of 10 eligible RD students consented to participate, with participant demographics summarized in Table 1 .

**Table 1:** Participant demographics by program

Demographic	SLP students ( *n* = 23)	RD students ( *n* = 8)	Total ( *N* = 31)
Age	M = 22.8 (21–26)	M = 24.6 (21–40)	M = 23.3 (21–40)
Gender			
Female	22 (95.7%)	8 (100%)	30 (96.8%)
Male	1 (4.3%)	0 (0%)	1 (3.2%)
Race a			
Black or African American	1 (4.3%)	0 (0%)	1 (3.2%)
White or Caucasian	22 (95.7%)	8 (100%)	30 (96.8%)
Ethnicity b			
Not Hispanic or Latino	22 (95.7%)	8 (100%)	30 (96.8%)
Disability status			
No reported disability	21 (91.3%)	8 (100%)	29 (93.5%)
Neurodivergence	2 (8.7%)	0 (0%)	2 (6.5%)

Abbreviations: M, mean; RD, registered dietitian; SLP, speech-language pathologist.

Notes: Race and ethnicity were reported separately.

a One participant reported biracial status.

b One participant did not report ethnicity.

### Procedures

The IPE sequence in this study consisted of two scenario and case-based learning sessions delivered within a single academic semester ( Table 2 ). Each session was 1 hour in length, with the first being held virtually on Zoom and the second being held in person beginning in a standard classroom. The instructors assigned students to groups of three or four, with two or three SLP students and one dietetics student per group. Before the first IPE session, students were required to watch a single video filmed by each course instructor introducing them to each profession. Each video was approximately 10 minutes long and overviewed the profession, including standard roles and responsibilities, required education and credentials, and common practice settings. These videos were used to establish baseline role awareness of the professions consistent with best practices in healthcare simulations (Chan et al., 2023; Penn et al., 2023).

**Table 2:** Timeline of interprofessional education activities

Week of semester	Activity	Purpose, key components, and learning objectives
5 (Asynchronous)	IPE Pre-Survey	**Purpose:** Establish baseline perceptions of IPE and role understanding.
6 (Asynchronous)	Videos introducing professions of SLP and RD (≈10 minutes each)	**Purpose:** Establish foundational role awareness prior to interprofessional interaction. **Learning objectives:** (a) Describe the education, credentials, and scope of practice of SLPs and RDs; (b) identify primary roles and responsibilities of each profession in dysphagia management.
7 (Asynchronous)	Pre-briefing for IPE Experience 1	**Purpose:** Prepare students for structured interprofessional interaction. **Learning objectives:** (a) Review expectations for interprofessional communication; (b) identify profession-specific concepts to be explained to professional colleagues.
8 (Synchronous)	IPE Experience 1 (virtual) with large-group debriefing	**Purpose:** Develop role clarity and interprofessional communication skills. **Learning objectives:** (a) Articulate profession-specific roles and responsibilities in dysphagia care; (b) communicate professional concepts clearly to interprofessional colleagues; (c) engage in respectful, collaborative dialogue around shared clinical concerns.
12 (Asynchronous)	Pre-briefing for IPE Experience 2	**Purpose:** Prepare students for applied interprofessional decision-making. **Learning objectives:** (a) Review patient-centered decision-making principles; (b) prepare to integrate professional knowledge within a team-based clinical task.
13 (Synchronous)	IPE Experience 2 (in person) with small-group debriefing	**Purpose:** Apply IPCP to patient-centered clinical decisions. **Learning objectives:** (a) Collaborate with interprofessional colleagues to develop shared care decisions; (b) integrate swallowing safety, nutritional needs, and patient preferences into care planning; (c) demonstrate teamwork, shared responsibility, and ethical decision-making.
15 (Asynchronous)	IPE Post-Survey	**Purpose:** Evaluate changes in perceptions of IPE and role understanding following the IPE sequence.

Abbreviations: IPCP, interprofessional collaborative practice; IPE, interprofessional education; RD, registered dietitian; SLP, speech-language pathologist.

Both IPE sessions were designed in a similar manner, including a highly structured pre-briefing, sessions featuring small group work, and a facilitated debriefing customized to each session's learning goals. The pre-briefing for each session promoted students' preparation by providing an overview of the upcoming session, goals for the experience, resource links, specific case materials, and scripted scenarios with discussion points ( Supplementary Appendix A and Appendix B (online only)). Pre-briefing materials were first overviewed with students and then posted with their course materials 1 week prior to each session. Students were encouraged to further prepare ahead of each session by reviewing their materials and reflecting on how specific components related to their profession. The purpose and learning goals of each experience were also reviewed at the beginning of each session. Both IPE sessions were facilitated by the course instructors, a graduate research assistant, and the principal investigator. Facilitators were not actively involved in the small group discussions beyond monitoring time-on-task by actively moving between breakout rooms and facilitating timely movement through planned instructional activities.

The first IPE session was conducted virtually via Zoom and emphasized the IPEC domains of roles and responsibilities and communication (Interprofessional Education Collaborative, 2023). Each small group was tasked with learning to communicate with one another while discussing “red flags” in their profession related to dysphagia care, explaining key concepts and terms, and sharing how their respective fields evaluate patients with dysphagia using common patient care scenarios as a platform for small group discussions ( Supplementary Appendix A (online only)). These discussions occurred in individual breakout rooms.

The second IPE session conducted in person underscored the importance of teams and teamwork and values and ethics in making patient-centered decisions about food choices for patients who require a modified diet due to dysphagia (Interprofessional Education Collaborative, 2023). Pre-briefing materials included a simulated patient profile with relevant medical history and comorbidities, diagnostic results from an instrumental assessment of swallowing, and laboratory findings important to guiding nutritional care. The small groups' collaborative learning experience included a joint review of these clinical reports and medical information followed by combining their professional knowledge to decide on food options for the patient's meals ( Supplementary Appendix B (online only)). The provided facility menu included a range of food choices to further emphasize the importance of mutual respect and shared decision-making in team-based care that valued the patient's well-being and personal goals. Each small group was situated within individual classrooms, research laboratories, and the speech-language pathology clinic's therapy rooms to reduce distractions and facilitate discussion.

Debriefing was customized to each learning experience and intentionally designed as a primary mechanism through which students could consolidate their learning at the end of each IPE session. Debriefings were structured using an evidence-informed, facilitator-guided approach consistent with established debriefing frameworks in health professions education that emphasize psychological safety, reflective analysis, and transfer to future practice (Eppich & Cheng, 2015; Cheng et al., 2021; Barlow et al., 2024).

At the conclusion of the virtual IPE session, small groups returned from their breakout rooms and all students selected one learning goal for the session and wrote one to two sentences about their experience using Zoom's whiteboard feature, which was visible to all participants. One of the group facilitators then provided summarizing comments. The debriefing for the in-person experience was conducted in individual small groups and emphasized their engagement with the session's learning goals. Each group was tasked with summarizing how the group accomplished each goal or experienced unique challenges or opportunities in attempting to meet it. They were then required to write a one-sentence takeaway about their IPE experience to an IPE facilitator at the conclusion of the session. The debriefing format was intentionally differentiated across experiences to match the logistical constraints and learning goals of each. Specifically, large group debriefing was planned for the first session due to the time required to orient participants to the virtual environment and establish psychological safety at the outset of the sequence, as well as the session's emphasis on roles and communication as foundational competencies for the sequence. Because the second session focused on the IPEC competencies of teams and teamwork as well as values and ethics, small group debriefing was used to allow for sustained, group-specific reflection on the collaborative reasoning process.

### Data Collection and Analysis

Quantitative data were collected using the Student Perceptions of Interprofessional Clinical Education–Revised (SPICE-R; Dominguez et al., 2015), a validated 10-item scale assessing attitudes toward teamwork and team-based care, professional roles and responsibilities, and the perceived impact of IPCP on patient outcomes. Participants also responded to two study-specific items asking them to rate their understanding of the roles and responsibilities of RDs and SLPs related to dysphagia management. These two items used a 7-point Likert-type scale ranging from 1 (very low understanding) to 7 (very high understanding).

Qualitative data were collected through open-ended prompts in the same survey at both pre-test and post-test. At both time points, participants were asked to describe the roles and responsibilities of RDs and SLPs in dysphagia management and share any additional reflections on IPE. After the second IPE event, participants responded to two additional questions asking them to reflect on how their IPE experiences influenced their ability to engage in IPP and how the two IPE experiences might affect their future work with other professionals and client populations.

### Quantitative Analysis

Quantitative analyses were conducted using SPSS (Version 30) and the first author tested the assumptions of the general linear model to determine the appropriateness of using parametric statistics with this dataset (Field, 2018). There were no significant differences between RD and SLP students on SPICE-R scores at pre- or post-test, and the data were collapsed into a single group due to a lack of theoretical expectation for differential responses between RDs and SLPs. Paired samples *t* -tests were subsequently used to evaluate differences on SPICE-R outcomes before and after the IPE sequence, with corresponding effect sizes (Cohen's *d* ) and confidence intervals. A Holm correction was applied across the four paired *t* -tests (SPICE-R total, teams and teamwork, roles and responsibilities, and patient outcomes) to control the familywise error rate. However, ratings of perceived knowledge of SLP and RD roles did not meet assumptions for parametric statistics and, as expected, differed significantly between professions at both time points. Data for these two items were thus analyzed separately by profession using Wilcoxon signed-rank tests, with effect sizes ( *r* = Z/√n) reported as point estimates not conventionally associated with a confidence interval. A Holm correction was also applied across the four tests to control the familywise error rate.

### Qualitative Analysis

Reflexive thematic analysis (Braun & Clarke, 2006; Braun & Clarke, 2021) was used to explore participants' reflections before and after the IPE sequence. The first and third authors independently familiarized themselves with the full dataset through repeated readings and inductive, analytic notetaking. Initial coding was conducted using NVivo (Version 15), with initial codes generated at the level of meaning units while remaining closely grounded in participants' language. The first and third authors used an inductive approach to coding in which patterns of meaning were constructed through sustained, iterative, and active engagement with the dataset over a period of several months.

Following initial coding, the first and third authors iteratively organized codes into potential themes, repeatedly evaluating emerging patterns within the dataset. Themes were developed through ongoing dialogue, analytic memoing, and reflexive discussions during weekly meetings. Candidate themes were refined until they demonstrated internal coherence, conceptual distinctiveness, and alignment with the overall analytic narrative.

Rigor was conceptualized in alignment with contemporary reflexive thematic analysis standards, emphasizing methodological coherence, reflexive engagement, and analytic transparency (Braun & Clarke, 2021; Braun & Clarke, 2023). Reflexive conversations were used to examine how researcher assumptions, professional training, and proximity to the instructional context shaped interpretation. The research team intentionally engaged with cases that diverged from dominant patterns to clarify the boundaries and complexity of emerging themes. Rich description and participant excerpts were used to illustrate and substantiate analytic claims during the theme development process.

### Researcher Positionality

The research team also explicitly considered how their identities and roles shaped analytic engagement during study design, implementation, and analysis (Folkes, 2023). The first three authors are faculty in the SLP program while the fourth author serves as program chair for the RD program. The second and fourth authors also served as instructors of record for the courses from which participants were recruited. Although these authors had the most direct contact with participants, the first and third authors were primarily responsible for data collection, analysis, and reporting.

The research team engaged in ongoing reflexive dialogue to examine how faculty–student power dynamics, professional perspectives, and commitments to IPE might influence data interpretation. Procedural safeguards were implemented to reduce coercion and protect participants' autonomy, including the use of grading rubrics during the semester and collection of informed consent by the first author when instructors of record were not present. These steps supported ethical rigor while reflexive practices supported interpretive transparency.

### Data Integration

Following separate analyses of quantitative and qualitative strands, data were integrated using a connecting and weaving approach (Fetters et al., 2013). Quantitative findings documented changes in students' self-reported perceptions of IPE and IPCP, and qualitative themes were then examined to deepen understanding of these shifts by exploring how participants described evolving conceptions of professional roles, experiences with collaboration, and anticipated application of what they learned from the IPE sequence to their own future clinical practice.

Points of convergence and divergence between the quantitative and qualitative strands were intentionally examined to inform overall interpretation of the results. The process of integrating the two strands was intentionally used to construct a more nuanced account of how participants experienced and interpreted changes in their self-reported perceptions of IPE and IPCP.

## Results

This study evaluated the impact of an IPE sequence on graduate students' perceptions of IPE, IPCP, and their self-reported understanding of professional roles in dysphagia management. Descriptive statistics indicated consistent upward shifts across all outcomes from pre- to post-IPE ( Table 3 ). Mean SPICE-R total scores increased from 39.74 (SD = 3.11) to 44.61 (SD = 2.86), with parallel gains observed in teams and teamwork (17.81 to 19.06), roles and responsibilities (9.77 to 12.26), and patient outcomes (12.16 to 13.29); median scores similarly increased and approached the upper range of the scale at post-test. Self-rated knowledge of SLP and RD roles also increased across professions, with post-test means ranging from 5.35 to 6.87 on the 7-point scale, reflecting substantial perceived gains in role clarity and interprofessional understanding.

**Table 3:** Descriptive statistics pre– and post–interprofessional education sequence

Outcome	Pre-IPE					Post-IPE			
	Min	Mdn	Mn (SD)	Max		Min	Mdn	Mn (SD)	Max
SPICE-R domain									
Teams and teamwork	15	18	17.81 (1.54)	20		15	20	19.06 (1.29)	20
Roles and responsibilities	7	9	9.77 (1.63)	14		9	12	12.26 (1.46)	15
Patient outcomes related to IPCP	9	12	12.16 (1.32)	15		10	13	13.29 (1.10)	15
Total	33	39	39.74 (3.11)	45		39	44	44.61 (2.86)	50
Roles and responsibilities of RDs									
RD	3	5	4.88 (0.99)	6		5	6	6.25 (0.71)	7
SLP	2	4	3.83 (1.37)	7		4	5	5.35 (0.78)	7
Roles and responsibilities of SLPs									
RD	2	4	3.38 (1.19)	5		4	5.5	5.50 (0.93)	7
SLP	4	6	5.78 (0.80)	7		6	7	6.87 (0.34)	7

Abbreviations: IPCP, interprofessional collaborative practice; IPE, interprofessional education; RDs, registered dietitians; SLPs, speech-language pathologists; SPICE-R, Student Perceptions of Interprofessional Clinical Education–Revised.

### RQ1: Quantitative Changes in Student Perceptions

Paired samples *t* -tests indicated statistically significant increase in SPICE-R scores following the IPE sequence. Total SPICE-R scores increased from pre- to post-test (t(30) = 9.15, *p* < 0.001), with a very large effect size (Cohen's d = 1.64, 95% CI [1.09, 2.18]). Significant improvements were also observed across all SPICE-R domains, including teams and teamwork (t(30) = 3.96, *p* < 0.001, d = 0.71, 95% CI [0.31, 1.10]), roles and responsibilities (t(30) = 8.09, *p* < 0.001, d = 1.45, 95% CI [0.94, 1.95]), and patient outcomes (t(30) = 5.22, *p* < 0.001, d = 0.94, 95% CI [0.51, 1.36]). All results remained statistically significant following Holm correction across the four tests. All effect sizes met or exceeded Cohen's (1988) benchmark for large effects (d ≥ 0.80), with the exception of teams and teamwork, which reflected a moderate-to-large effect.

At each time point, participants also rated their perceived knowledge of the roles and responsibilities of SLPs and RDs in dysphagia management using a 7-point Likert-type scale. These data did not meet assumptions for parametric testing and differed significantly by profession at both time points. Accordingly, Wilcoxon signed-rank tests were conducted separately by profession. Among SLP participants, self-ratings of knowledge of SLP roles increased significantly from pre- to post-test ( *p* < 0.001, r = 0.80). Similarly, SLP self-ratings of knowledge of RD roles increased significantly ( *p* < 0.001, r = 0.75). Among RD participants ( *n* = 8), self-ratings of knowledge of SLP roles increased significantly ( *p* = 0.011, r = 0.90). RD participants also demonstrated significant gains in their understanding of RD roles ( *p* = 0.026, r = 0.79). All results remained statistically significant following Holm correction across the four tests, with all effects exceeding conventional benchmarks for large effects (r > 0.50).

### RQ2: Qualitative Changes after IPE and Looking Ahead to the Future

Reflexive thematic analysis generated an interpretive account of how participation in dysphagia-focused IPE reshaped students' understandings of their professional roles and future practice. Participants described drawing sharper professional boundaries, negotiating expertise in real time within IPE experiences, and reframing dysphagia management such as sharing patient risk in ways that require integrated clinical reasoning. Students positioned the experience as rehearsal for future clinical opportunities and reflected on how it influenced their developing sense of themselves as clinicians working within an interdependent healthcare system. Together, these themes suggest that the IPE sequence operated as a context for boundary clarification, collaborative reasoning, and early negotiation of their professional identities.

#### Theme 1: Moving from Broad Descriptions to Specific Roles

Before the IPE sequence, participants described the professional responsibilities of RDs and SLPs in broad terms. For example, one SLP participant said, “SLP's [sic] evaluate, diagnose, treat, educate, and refer patients that have impaired swallowing or feeding” (SLP-18) while an RD participant wrote, “... [we] ensure the diet is appropriate for the patient” (RD-3). After completing the IPE activities, participants' descriptions of roles and responsibilities included more profession-specific terminology and more descriptive details. For instance, SLP-17 explained that RDs evaluate “lab results, observations, and formal/informal assessments of a client's diet and nutritional health” while RD-5 noted that SLPs assign patients “an [International Dysphagia Diet Standardisation Initiative] level... and communicates needs to the rest of the interprofessional team.” After the IPE experience, participants frequently referenced profession-specific assessments and techniques, including nutrition-focused physical examinations (NFPE), videofluroscopic studies (VFSS), and fiberoptic endoscopic evaluations of swallowing (FEES). Notably, this expansion and increased specificity extended across professional boundaries as participants articulated not only their own responsibilities, but also the processes and tools central to their colleagues' role in dysphagia management.

#### Theme 2: Negotiating Unfamiliar Terrain Across Professions

Before the first IPE activity, participants described IPE and IPCP in vague, nonspecific terms such as when SLP-11 wrote “IPE helps professionals learn.” After completing the IPE activities, participants described collaboration in more situated and specific ways, emphasizing interdependence, shared goals, and active communication across professional boundaries. For example, RD-7 shared, “I now have a better understanding of how RDs and SLPs work together, improved my communication skills for effective teamwork, and saw firsthand how patient-centered care benefits from a holistic approach.” Similarly, SLP-11 wrote, “I have developed a collaborative mindset that prioritizes teamwork and shared decision-making.” Beyond attitudinal shifts, participants described specific strategies used during the IPE experiences, including negotiating terminology, clarifying assumptions, and adapting language to reduce misunderstandings. For example, SLP-15 wrote, “I learned that each profession has their own terminology, so as a professional, asking questions when you don't understand or fully know will help in the collaboration and the client's evaluation and treatment.” RD-1 also captured this sense of productive difference, writing that “a dietitian's view of care looks different than a SLP's” and that the groups “capitalized on our different strengths.” This theme reflects patterns of meaning associated with an active process of collaboration requiring ongoing clarification and mutual adjustment across professional boundaries.

#### Theme 3: Framing Dysphagia as Sharing Patient Risk

Participants described a shift in how they conceptualized caring for patients with dysphagia, specifically moving from profession-centered outputs to a more holistic, integrated consideration of patient risk and health outcomes. Before the IPE sequence, participants made general statements about individualized care plans, such as when RD-4 wrote, “SLPs generate an individualized care plan that focuses on enhancing their swallowing efficiency.” These pre-IPE descriptions acknowledged that care plans exist, but did not elaborate on how patient safety, nutritional adequacy, or physiological stability could be jointly considered across professions.

In contrast, after the IPE sequence participants better articulated how each profession incorporated multiple sources of data and identified overlap between professional responsibilities. For example, SLP-5 wrote that RDs were responsible for monitoring “client bloodwork and test reports, modifying diets to support/lead to more healthy lifestyles, providing clients with increased nutritional meals, monitoring their test work levels, and seeing that the clients are progressing towards a health weight, glucose levels, etc.” RD-4 observed that “the Speech-Language Pathology students selected foods primarily based on their safety for swallowing, while my approach centered on addressing the patient's underlying medical conditions, such as diabetes or hypertension,” concluding that this contrast “highlighted the necessity of interdisciplinary collaboration to develop care plans that prioritize both the safety and overall health of the individual.” Rather than describing isolated professional tasks, participants emphasized how dietary modifications, swallow safety recommendations, laboratory values, and overall health status intersected in dysphagia management.

#### Theme 4: Rehearsing for Anticipated Collaborations

After the IPE sequence, participants described specific ways in which the activities extended beyond their immediate learning and toward their future clinical practice. Before the first IPE event, participants primarily expressed a vague hope that IPE would be helpful to them, which was consistent with their nonspecific descriptions of what they thought IPE was. After completing the IPE activities, participants described the experience as anticipatory rehearsal for real-world collaboration. One participant reflected, “I am so grateful we had this experience in class ... instead of having to first experience it in the workplace” (SLP-21), explicitly contrasting classroom exposure with first-time workplace encounters. RD-5 also shared that the IPE events had left them with a newfound knowledge of how the two professions overlap and that, as a result, “now I know what patient information I might need to explain to an SLP in the future.”

Similarly, SLP-16 noted, “We worked together to find the best ‘fit’ for our patient, and I would like to think this won't be the only time doing so,” signaling expectation of future collaborative engagement. RD-3 also wrote, “this opportunity has made me so eager to dive into our clinical rotations and expand my knowledge with how to interact with SLPs.” Post-IPE reflections emphasized the experience as rehearsal space in which they could simulate and practice IPCP before entering independent clinical environments.

#### Theme 5: Repositioning the Self Within an Interprofessional Identity

Participants also reported that the IPE sequence refined their understanding of the roles and responsibilities associated with their own profession, including their role on an interprofessional team. Many participants expressed uncertainty about their preparedness for IPCP prior to the IPE sequence. For example, RD-8 wrote, “I don't feel that I know enough yet!,” suggesting perceived gaps in their existing training and the clinical expectations of the professions. However, after the IPE sequence participants described a stronger alignment between their curricular training experiences and their future professional practice, such as when SLP-15 shared, “it enhanced my understanding and knowledge of my future scope of practice,” and SLP-1 wrote, “it allowed me to combine both my clinical knowledge and my professional abilities.” This theme emphasizes participants' efforts to reorient themselves within their emerging sense of professional identity, which is distinct from the procedural shifts associated with Theme 1.

### RQ3: Qualitative Expansion on Quantitative Findings

Quantitative findings demonstrated statistically significant and large improvements in SPICE-R total scores and across all subdomains, as well as profession-specific increase in perceived knowledge of roles and responsibilities. The qualitative themes provided interpretive depth regarding how participants experienced and interpreted these shifts, and the integration of both strands is displayed in Table 4 .

**Table 4:** Joint display of quantitative and qualitative findings

Quantitative outcome	Qualitative theme	Pre-IPE excerpt	Post-IPE excerpt	Meta-inference
SPICE-R: Roles and responsibilities	Theme 1: Moving from broad descriptions to specific roles	“...[we] ensure the diet is appropriate for the patient” (RD-3).	“The RD ensures that the patient's diet is meeting the [International Dysphagia Diet Standardisation Initiative] level that the SLP assigned to the patient, along with meeting standards for other chronic diseases the patient might have...” (RD-5).	Quantitative gains in role clarity corresponded with participants' shift from generalized role descriptions to using profession-specific terminology.
SPICE-R: Teams and teamwork	Theme 2: Negotiating unfamiliar terrain across professions	“IPE helps professionals learn…” (SLP-11).	“Working through the case studies with the SLP students showed me the value of combining expertise to solve complex problems” (RD-7).	Improvements in teamwork perceptions corresponded with qualitative evidence of active, strategy-driven collaboration requiring ongoing adjustment of communication.
SPICE-R: Patient outcomes	Theme 3: Framing dysphagia as sharing patient risk	“SLPs generate an individualized care plan that focuses on enhancing their swallowing efficiency” (RD-4).	“The SLP students selected foods primarily based on safety for swallowing, while my approach centered on the patient's underlying medical conditions...this contrast highlighted the necessity of interdisciplinary collaboration” (RD-4).	Gains in perceived impact on patient outcomes were elaborated qualitatively as a shift from describing profession-centered task to more integrated, shared management of patient risk.
Total SPICE-R	Theme 4: Rehearsing for anticipated collaborations	“I think IPE will be an applicable experience that will help prepare us” (SLP-9).	“I am so grateful we had this experience in class...instead of having to first experience it in the workplace” (SLP-21).	Improvements in total SPICE-R scores were elaborated as anticipatory rehearsal, with participants framing the IPE sequence as preparation for future collaborative practice they would otherwise first encounter in the workplace.
Self-ratings of role knowledge (SLP and RD)	Theme 5: Repositioning the self within an interprofessional identity	“I don't feel that I know enough yet!” (RD-8).	“I have learned what it looks like to understand another's profession, and how I can approach this opportunity as an experience to learn more, for me and my client” (SLP-23).	Gains in participants' self-rated knowledge of SLP and RD roles were accompanied by a qualitative repositioning of professional identity, as students situated their developing role knowledge within an emerging interprofessional sense of self.

Abbreviations: RD, registered dietitian; SLP, speech-language pathologist; SPICE-R, Student Perceptions of Interprofessional Clinical Education–Revised.

Note: Quantitative outcomes include SPICE-R subscale scores, the total SPICE-R score, and profession-specific self-ratings of role knowledge. Excerpts are illustrative; participant identifiers denote profession (SLP, RD) and participant number.

Improvements within the SPICE-R roles and responsibilities domain corresponded with the theme moving from broad descriptions to specific roles, in which participants articulated expanded, profession-specific understandings of both their own and their colleagues' professional contributions to dysphagia care. Similarly, gains within the teams and teamwork domain aligned with negotiating unfamiliar terrain across professions, as participants described collaboration as an active process involving clarification, shared reasoning, and communicative adjustment. Increase within the patient outcomes domain paralleled framing dysphagia as sharing patient risk, where participants emphasized integrating considerations related to swallowing safety, nutritional adequacy, and physiological stability.

Although the first three themes correspond well with the individual domains of the SPICE-R, rehearsing for anticipated collaborations and repositioning the self within an interprofessional identity extended the quantitative findings associated with individual SPICE-R domains by illuminating participants' anticipatory readiness and evolving professional identity within collaborative healthcare systems. Across the quantitative and qualitative strands, no substantive contradictions were identified. Instead, the qualitative findings elaborate on the quantitative outcomes by highlighting the processes through which students' perceptions, collaborative reasoning, and professional self-understandings shifted during the IPE sequence.

## Discussion

This mixed methods study examined whether a two-part, dysphagia-focused IPE sequence embedded within graduate training could meaningfully influence students' perceptions of IPCP and their understanding of shared clinical responsibility. Quantitative findings demonstrated statistically significant, large improvements across all SPICE-R domains and profession-specific role knowledge items. Qualitative findings clarified how these shifts occurred, revealing movement in students' perceptions from generalized ideals to technically grounded, role-specific reasoning and shared framing of patient risk in dysphagia management. Together, these findings correspond closely with key IPEC competencies, particularly roles/responsibilities and interprofessional communication, which emphasize clarity in professional scope and effective communication in collaborative clinical decision-making (Interprofessional Education Collaborative, 2023). The present study's outcomes are consistent with the structure of the two IPE experiences themselves whereby the first activity emphasized clarifying professional roles and communicating profession-specific concepts and the second required students to apply that knowledge collaboratively when making patient-centered decisions about diet modification and nutritional management.

The present findings are consistent with systematic reviews demonstrating that IPE most reliably improves early-level outcomes such as attitudes toward collaboration, understanding of professional roles, and perceived readiness for team-based care, whereas behavioral and patient-level outcomes are more challenging to measure (Spaulding et al., 2021; Saragih et al., 2023; Mattiazzi et al., 2024). The magnitude of the present SPICE-R gains aligns with prior reports of short, case-based, or simulation-enhanced IPE experiences producing measurable changes in trainees' perceptions and confidence (Matulewicz et al., 2020; Naumann et al., 2021). However, although moderate-to-large effects were observed in both SLP and RD participants, RD-specific findings related to role knowledge should be interpreted as exploratory given the small size of their subsample ( *n* = 8).

One interpretation of these findings is that the IPE sequence functioned as an exercise in collaborative clinical reasoning. Dysphagia management requires negotiating professional priorities, including aspiration risk, nutritional adequacy, laboratory values, and feasibility of recommendations for the patient and the care team. IPCP has been defined as the process by which professionals jointly interpret clinical information and reconcile profession-specific priorities to reduce uncertainty and improve outcomes in patient care (Kiesewetter et al., 2017). Qualitative patterns related to negotiating communication between professions, integrating laboratory data with instrumental findings, and framing dysphagia as a risk to the patient shared by multiple providers suggest that students were going beyond improving their attitudes toward teamwork to engage in early forms of negotiated clinical reasoning across professional boundaries. This pattern reflects the design of the second IPE experience, which required students to integrate instrumental swallowing findings, nutritional indicators, and patient preferences while negotiating diet recommendations in small interprofessional teams.

Dysphagia provides a uniquely appropriate clinical context for IPE because it inherently requires coordinated decision-making that integrates physiologic safety, nutritional management, and quality of life (McGinnis et al., 2019). Unlike some interprofessional contexts where roles may remain parallel, dysphagia management often requires real-time negotiation of diet modifications, texture standards, laboratory findings, and instrumental data on swallowing function.

The present study extends the existing literature by identifying interpretive changes in how students conceptualize shared risk and responsibility related to dysphagia in addition to previously documented improvements in attitudes toward IPCP. Existing dysphagia-focused IPE studies have demonstrated improvements in learner confidence and preparedness (Paramby et al., 2019; Nandamudi et al., 2023) but have not integrated quantitative measures of perception with qualitative analysis to clarify how trainees' reasoning processes shift.

The present study also extends the existing literature through the emergence of identity-related patterns in the dataset despite not intentionally designing the IPE sequence to function as a professional identity intervention. Current literature on professional identity development suggests that participating in authentic, practice-based collaboration can contribute to the development of professional identities within an interprofessional schema (Sarraf-Yazdi et al., 2021; Grymonpre et al., 2023). The present findings suggest that dysphagia-focused IPE may serve as an early context in which students can begin reconciling their personal competence, professional scope, and accountability to other members of an interprofessional team.

### Implications for Education and Practice

These findings support considering how to embed IPE activities within existing coursework instead of creating standalone events without a conceptual anchor to ongoing learning. Even brief, well-structured activities that require interdependence, case-based problem solving, and guided debriefing may meaningfully influence how trainees conceptualize shared responsibility for high-risk medical conditions. Incorporating intentional pre-briefing may further strengthen these experiences by preparing students to engage productively in the interprofessional encounter, allowing faculty to maximize the value of relatively brief IPE activities without requiring extensive additional class time (Chan et al., 2023; Penn et al., 2023). This approach may be particularly important for programs facing scheduling constraints, where longer interprofessional experiences are difficult to coordinate across professions. For dysphagia in particular, early exposure to interprofessional reasoning may reduce fragmented decision-making in future practice by reinforcing explicit connections between swallowing safety, nutrition management, and feasibility of implementation. Future curricular efforts can also benefit from explicitly aligning IPE experiences to target specific IPEC competencies, particularly roles/responsibilities and interprofessional communication, to ensure that activities support the progressive development of IPCP.

## Limitations

Although the SPICE-R is a validated and widely used instrument for assessing student perceptions of IPE (Dominguez et al., 2015), self-report instruments primarily measure perceived competence and attitudes rather than observed collaborative behaviors, with variable coverage and ability to predict future clinical performance (Bainbridge et al., 2023; Mattiazzi et al., 2024). Additionally, pre-test means on the SPICE-R were already high, suggesting potential ceiling effects dampening the magnitude of observed gains. Maturation effects also cannot be ruled out given the nonexperimental design. The absence of follow-up testing precludes concluding that immediate gains in perceived competence post-IPE transfer to clinical practice and represents an important consideration for future research. Future work should also incorporate behavioral measures of communication and collaborative decision-making during IPE activities or in subsequent clinical placements to examine transfer beyond student perception.

It is also important to consider the 23:8 ratio of SLP to RD students, which reflects the relative enrollments of the participating programs. Although RD perspectives are represented across all themes, the findings should be contextualized as more densely supported by data featuring SLP voices. Relatedly, because each small group included two or three SLP students for every one RD student, RD students' experience of group dynamics and discussion patterns during the IPE sessions may have differed from that of their SLP peers. Future research should more directly examine how group ratios impact students' perspectives on IPE and explore more immediate reactions to IPE events. Furthermore, because large-group debriefing was used at time point one while small-group debriefing occurred at time point two, it is possible that this change influenced not only participants' experiences but also the content of their written reflections that informed the qualitative strand of this study. The small RD subsample similarly limits the precision of the profession-specific quantitative estimates, and the RD subgroup findings should be treated as exploratory pending replication with a larger sample. Participants were also recruited from a single institution using a nonexperimental design that prevents concluding changes in student perceptions and perspectives were caused by the IPE experiences themselves. SLP students' enrollment in concurrent dysphagia coursework and/or RD students' enrollment in non-acute care clinical rotations could have influenced the present findings. Finally, despite the procedural safeguards reported in the Methods, social desirability bias cannot be excluded given the overlap between researcher and instructor roles in the present study.

## Data Availability

The dataset used in the current study is not publicly available due to the personal and potentially identifiable nature of participants' reflections and because consent for public data sharing was not obtained. De-identified excerpts relevant to the study findings are included within the article and additional de-identified data may be available from the corresponding author upon reasonable request.

## Declaration of GenAI Use

Artificial intelligence tools (ChatGPT for the initial manuscript and Claude for subsequent revisions) were used during manuscript preparation to support text editing and refine the language used to communicate ideas. All content in the manuscript was reviewed, revised, and approved by the authors before submission.
