# Supplementary material for: Interprofessional Education with Graduate Students in Dietetics and Speech-Language Pathology: A Sequential-Explanatory Investigation
Source: Semin Speech Lang. 2026 Jul 28;47(3):138–48. doi: 10.1055/a-2914-5938 (PMC13614848; doi:10.1055/a-2914-5938)
Supplement: Supplementary file 1 [file 10-1055-a-2914-5938-s260006resa-a.pdf]

Appendix A

Interprofessional Education Experience: Emphasis on IPEC Core Competencies of *Roles and Responsibilities* and *Communication*

Overview:

The IPE experience integrates professionals from Communication Sciences and Disorders (Speech-Language Pathologists; SLPs) and Dietetics (Registered Dietitians; RDs) as it relates to patient/client care. It will be virtual. After a brief welcome, you will be assigned to small groups by the course instructors. Each group will have a graduate learner from the dietetics program with 2-3 communication sciences and disorders (CSD) graduate learners. An instructor/IPE observer will join your group for brief periods during the activity. The IPE experience will be about one hour in duration.

You are encouraged to <u>prepare ahead of time</u>. That is, review this activity and think about how you might respond to specific aspects representing your profession <u>PRIOR</u> to the IPE session.

Goals of the IPE Experience:

1. Establish a working relationship with group members (professional colleagues)
2. Explain the significance of a new concept or term as it relates to patient care
3. Communicate clearly about professional concepts as a speaker and listener

An important component is to build and share professional knowledge with one another about common terms and concepts when providing patient care. Listed below are the specific concepts/terms you will be discussing/applying to situations during small group interactions.

CSD learners (representing speech-language pathologists):

- Red flags (for concerns about swallowing safety)
- Instrumental assessment (MBS vs. FEES)
    - Penetration Vs. Aspiration
- Oral dysphagia Vs. Pharyngeal dysphagia

Dietetics learners (representing registered dietitians):

- Red flags (for nutritional concerns)
    - Lab Values (protein), Weight, Comorbidities
- Nutrition Focused Physical Exam (NFPE)/Malnutrition Screening
- ADIME (nutrition care practice)

IPE Process and Procedures

Once assigned to a small group, spend the first few minutes introducing yourself to one another (about 1-2 minutes per person). Briefly indicate why you selected your profession, current level of training, and/or professional interest. You are all responsible for being considerate of time.

Seminars in Speech and Language | DOI 10.1055/a-2914-5938 | © The Author(s).

Please progress through your collaborative learning exercise in the following sequence:

Scenario 1: Red Flags (about 15 minutes)

You are part of an interprofessional team at an acute care hospital or inpatient rehabilitation setting who frequently evaluate people who have a diagnosis of stroke, disease, or traumatic brain injury. At a team meeting, you are asked to educate and inform new staff (e.g., nurses, therapists, case managers) about "red flag" swallowing or nutrition concerns for new patient admits.

Each profession will overview "red flag" concerns as they relate to their profession of practice. Allow about 3-5 minutes for each profession. Be sure your explanations clarify the importance of "red flags" for unfamiliar team members.

<u>Small Group Discussion:</u>

- First, clarify any areas/terms of confusion or uncertainty about red flags/related topics.
- In this scenario, you were asked to communicate information about "red flags" to <u>new staff</u>.
    - Dietetics learners – Discuss and ask questions about 2-3 red flags mentioned by the SLP that you found most impactful and consequential to effective patient care.
    - CSD learners – Discuss and ask questions about 2-3 red flags mentioned by the dietitian that you found most impactful and consequential to effective patient care.

Provide ongoing feedback to one another during your discussion. For example, if you do not understand what a colleague is saying about a "red flag" concern, ask for clarification in a professional manner. Alternatively, a colleague's feedback or explanation of a red flag concern may reflect misinformation. Offer a different suggestion or way to explain to new staff. In short, please provide supportive or corrective feedback as it is an essential aspect of patient/client care for both professions.

Scenario #2: Assessment (about 10 minutes)

An evaluation of one's status is a critical aspect of patient care. In this scenario, the nursing staff at your medical setting is responsible for scheduling and coordinating different assessments. Start this scenario by overviewing assessment themes with one another. Describe assessments in a manner that is relevant and understandable to <u>nursing staff</u>. Allow about 3-5 minutes for each profession.

- A CSD student will overview the basics of instrumental assessment (MBS vs. FEES), including what they represent and primary purposes. As part of this overview, clarify terms such as penetration and aspiration.
- Nutrition Focused Physical Exam (NFPE)/Malnutrition Screening will be overviewed by a dietetics learner.

<u>Small Group Discussion:</u>

What are likely clarification questions or follow-up questions that your nursing staff will ask about assessment procedures?

- Each group member should be able to <u>ask</u> a clarification question or follow-up question that may be of importance to nursing staff.
- Each group member should be able to <u>answer</u> a clarification question or follow-up question about one assessment procedure.

Seminars in Speech and Language | DOI 10.1055/a-2914-5938 | © The Author(s).

Provide ongoing feedback to one another during your discussion. For example, if you do not understand what a colleague is saying, ask for clarification in a professional manner. Alternatively, a colleague's feedback or explanation may reflect misinformation. Offer a different suggestion or way to explain a concept to nursing staff. In short, please provide supportive or corrective feedback as it is an essential aspect of patient/client care for both professions.

Scenario #3: Diagnostic Information or Outcomes (about 15 minutes)

It is common for patients and families to have many questions about health conditions and diagnoses. As health care providers, we often answer general questions posed by patients/families given our basic knowledge about a topic. Begin this scenario by describing/explaining terms with one another (3-5 minutes each):

- A CSD student will explain/define what is meant by a diagnosis of oral dysphagia versus pharyngeal dysphagia.
- A dietetics learner will overview the meaning of ADIME and how this process informs nutrition care.

<u>Small Group Discussion:</u>
Spend the first few minutes of your small group discussion clarifying concepts/terms as needed. Although SLPs and RDs strive to provide patients/families with clear communications, questions may persist. You may be the professional who is asked to clarify information from another area of practice.

- Dietetics learner - Imagine you enter a patient's room soon after a SLP has discussed a diagnosis of "oral dysphagia" with a patient and family. Their first questions to you include: "The speech person will be back tomorrow, but I have questions right now. Can you tell me more about it? I'm not sure I really understand what it all has to do with eating."
    - How would you generally describe a diagnosis of oral dysphagia to a patient/family?
    - Can you clarify how this diagnosis might affect the patient's nutrition (what the patient is able to eat or drink?)

- CSD learner – Imagine you enter a patient's room soon after a registered dietitian has completed their ADIME process. The patient and family indicate that the RD said, "given swallowing concerns, supplements might be needed to address energy and protein needs."
    - Based on your understanding of ADIME, what parts of the process might be informing this outcome?
    - How can you relate the family's comment to what you know about the patient's swallowing status?

Provide ongoing feedback to one another during your discussion. For example, if you do not understand what a colleague is saying, ask for clarification in a professional manner. Alternatively, a colleague's feedback or explanation of a new concept may reflect misinformation. Offer a different suggestion or way to explain a concept to a patient and their family. In short, please provide supportive or corrective feedback as it is an essential aspect of patient/client care for both professions.

Seminars in Speech and Language | DOI 10.1055/a-2914-5938 | © The Author(s).
