# Supplementary material for: Interprofessional Education with Graduate Students in Dietetics and Speech-Language Pathology: A Sequential-Explanatory Investigation
Source: Semin Speech Lang. 2026 Jul 28;47(3):138–48. doi: 10.1055/a-2914-5938 (PMC13614848; doi:10.1055/a-2914-5938)
Supplement: Supplementary file 2 [file 10-1055-a-2914-5938-s260006resa-b.pdf]

Appendix B

Interprofessional Education Experience: Emphasis on IPEC Core Competencies of *Teams and Teamwork* and *Values and Ethics*

Overview:

After a brief welcome, you will work in the small groups that were previously established. The IPE experience is approximately 1 hour. An instructor/IPE observer may observe your group for brief periods during the activity.

This experience builds on the knowledge you acquired about the professional roles and responsibilities of speech-language pathologists and registered dietitians in dysphagia management. The primary focus of this experience is a case presentation with discussion questions and activities that relate to it. The case study is a composite of patients illustrating swallowing and nutritional concerns, including representative medical chart information.

You are encouraged to <u>prepare ahead of time</u>. That is, review the case study and related resources, including a link to common medical abbreviations representative of what you might encounter in a digital medical chart in a health care environment, and the IDDSI framework documents for levels of diet modification. Think about how you might respond to specific aspects representing your profession <u>PRIOR</u> to the experience.

Goals of the IPE Experience:

1. Demonstrate an effective, collaborative working relationship with group members (professional colleagues).
2. Apply professional knowledge about diet modifications and nutritional health to a case study presentation of a patient who had a stroke.
3. Provide ongoing feedback, clarification, and clear communications to professional colleagues.

IPE Process and Procedures

This is an opportunity to interact with one another in a professional manner as future colleagues. Once you are gathered with group members, take a few minutes to touch base. Although you previously interacted virtually, this is your first face-to-face interaction. Small talk is a helpful tool in any professional environment.

Your case study collaborative learning exercise is divided into two parts:

Part 1: Review of Reports and Medical Information

Review and discuss the case study in your small group. Use the following topics to guide your small group interaction about the case:

Seminars in Speech and Language | DOI 10.1055/a-2914-5938 | © The Author(s).

- Are you familiar with various medical abbreviations and acronyms? Be sure to clarify what they represent with your group members. (Some may be more familiar with one profession vs. the other.)
- Each profession must provide a concise summation of their professional's report information. What is important to know? Communicate clearly about key concepts and their importance to patient care, including recommendations.
- What parts of the patient's history, background, and medical status were especially important to your profession of practice?
- As part of the first IPE experience, you learned about red flags. What, if any, red flags support resulting concerns in the patient's swallowing and the patient's nutritional well-being?

Provide ongoing feedback to one another during your small group interaction. For example, if you do not understand what a colleague is saying, ask for clarification in a professional manner. It is expected that all members will actively contribute to the small group discussion.

Part 2: Application of Professional Knowledge as Related to Diet Modifications

Now that you have reviewed the case study, including the patient's stated goal and food preferences, refer to the Menu for this part of the exercise. It represents a generic menu with options for each meal of the day. Use the menu in discussing the following:

- Select the "best" option(s) for each meal. What choices, if any, align with your patient's current dietary recommendations for safe swallowing (IDDSI Level 5) and nutritional needs (e.g., caloric, energy, protein needs)?
- Discuss and clarify why other food choices do not fit or represent undesirable choices.
- Are there ways to modify current food choices or alternative choices that might be more in line with current recommendations? Try to identify one change or suggestion for each meal.

Lastly, each group will be given samples of thickening products. Use the samples in discussing the following:

- What can you learn about each product based on its ingredients?
- In what way, if any, do thickening products change nutrient content or caloric intake?
- Are there any restrictions or considerations in thickening that relate to comorbidities such as diabetes or allergies?

Provide ongoing feedback to one another during your small group interaction. For example, if you do not understand what a colleague is saying, ask for clarification in a professional manner. It is expected that all members will actively contribute to the small group discussion.

Seminars in Speech and Language | DOI 10.1055/a-2914-5938 | © The Author(s).
